# Early sclerostin assessment in frail elderly patients with sepsis: insights on short- and long-term mortality prediction

**DOI:** 10.1007/s11739-023-03223-w

**Published:** 2023-03-21

**Authors:** Amedeo Tirandi, Eleonora Arboscello, Stefano Ministrini, Luca Liberale, Aldo Bonaventura, Alessandra Vecchié, Maria Bertolotto, Daniele Roberto Giacobbe, Luca Castellani, Michele Mirabella, Silvia Minetti, Matteo Bassetti, Fabrizio Montecucco, Federico Carbone

**Affiliations:** 1 Department of Internal Medicine, University of Genoa, 6 viale Benedetto XV, 16132 Genoa, Italy; 2 IRCCS Ospedale Policlinico San Martino, 10 Largo Rosanna Benzi, 16132 Genoa, Italy; 3 Center for Molecular Cardiology, University of Zurich, Wagistrasse 12, 8952 Schlieren, Switzerland; 4 Internal Medicine, Angiology and Atherosclerosis, Department of Medicine and Surgery, Università Degli Studi di Perugia, piazzale Gambuli 1, 06129 Perugia, Italy; 5 Medicina Generale 1, Medical Center, Ospedale di Circolo e Fondazione Macchi, ASST Sette Laghi, Varese, Italy; 6 Department of Internal Medicine, ASST Sette Laghi, Varese, Italy; 7 Department of Health Sciences (DISSAL), University of Genoa, Genoa, Italy

**Keywords:** Sepsis, Septic shock, Sclerostin, Inflammation, Mortality, Elderly

## Abstract

Unmet needs challenge clinical management of sepsis especially concerning patient profiling, enhancing recovery, and long-term sequelae. Here, we preliminarily focused on sclerostin (SOST) as a candidate biomarker to encompass such a broad range of clinical needs related to sepsis. Seventy-three septic patients were enrolled at internal medicine wards between January 2017 and December 2019 in this pilot study. Clinical examination and blood sample analyses were collected at enrollment and after 7 and 14 days. SOST levels were assessed on serum by ELISA. Thirty-day mortality was set as primary outcome. In-hospital and long-term mortality (2.5 years of median follow-up) were assessed as secondary outcomes. Patients were frail, elderly, and heterogeneous in terms of comorbidity burden. SOST levels were associated with age, cardiovascular comorbidities, and time to early death (30 days). When regression models were built, SOST displayed a high predictive value toward 30-day mortality (OR 13.459 with 95% CI 1.226–148.017) with ever better performance than validated scoring scales for critical ill patients. Such a predictive value of SOST was further confirmed for in-hospital (HR 10.089 with 95% CI 1.375–74.013) and long-term mortality (HR 5.061 with 95% CI 1.379–18.570). SOST levels generally decreased over 7 to 14 days after enrollment (*p* for trend < 0.001). The degree of this variation further predicted long-term mortality (HR for Δ SOST T0–day 14: 1.006 with 95% CI 1.001–1.011). Our results suggest a role for SOST in both short- and long-time prediction of worse outcome in septic elderly admitted to internal medicine wards.

**Graphical abstract:**

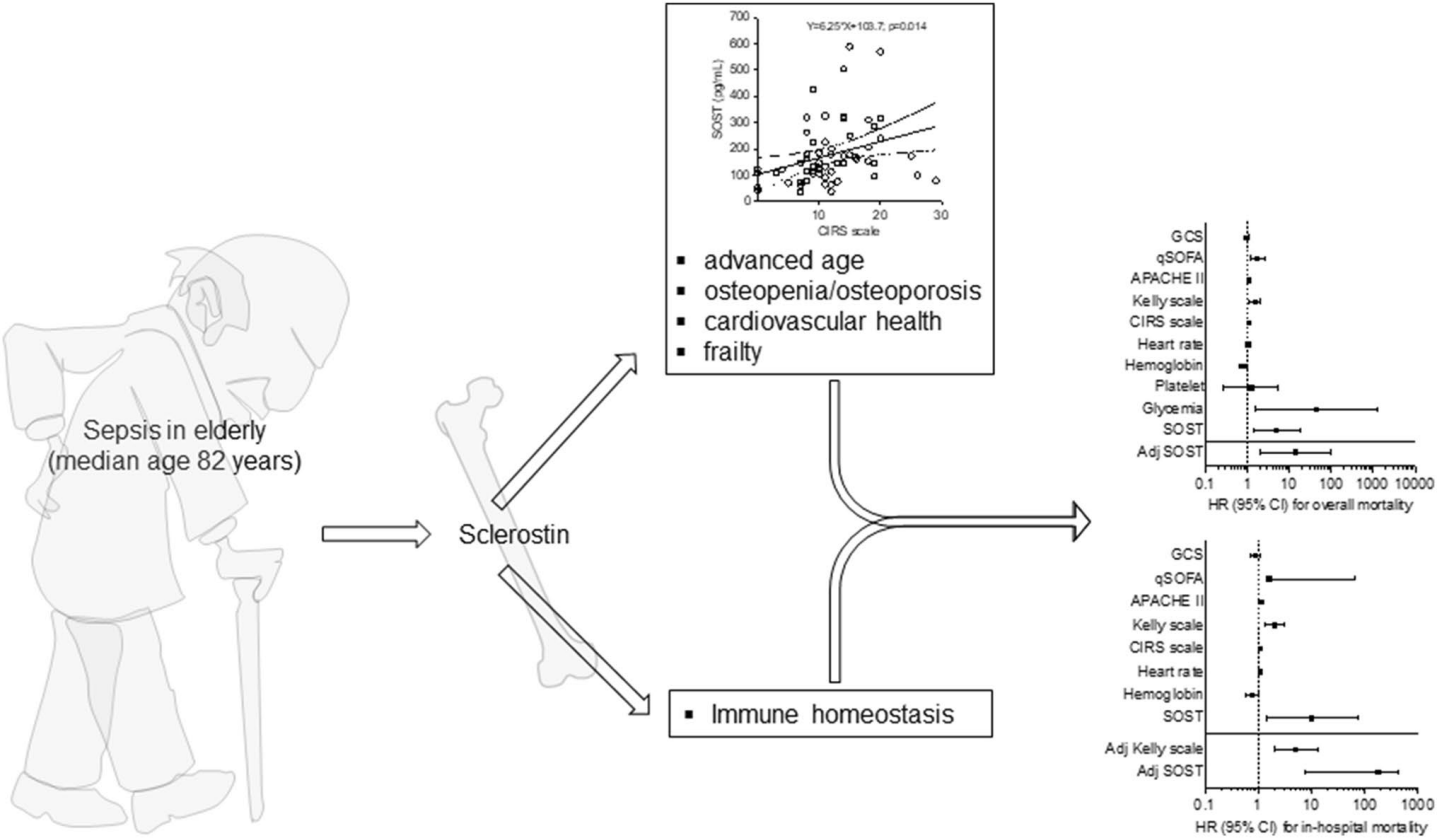

**Supplementary Information:**

The online version contains supplementary material available at 10.1007/s11739-023-03223-w.

## Highlights

- Septic elderly patients are at high risk of worse outcome.
- Sclerostin is marker of frailty and predictor of early and long-term mortality.
- Sclerostin is associated with sepsis-related mortality in elderly.

**Supplementary Information:**

The online version contains supplementary material available at 10.1007/s11739-023-03223-w.

## Introduction

The third international consensus definition of sepsis [1] upsets the traditional paradigm of this life-threatening condition. The shift toward dysregulated immune response as a leading determinant of organ damage has further highlighted the variability and complexity of host immune system and clinical outcome. Novel clinical phenotypes for sepsis with broad differences in clinical characteristics and organ dysfunction patterns are increasingly described [2]. They range from that with fewer, mild biochemical abnormalities, and organ dysfunction to those characterized by older age, greater comorbidity burden, and higher rate of renal impairment and shock. Inflammatory biomarkers may have a value in discriminating different phenotypes, but there are still far from a routine clinical application. However, incidence and costs of long-term sepsis-related mortality remain abysmal and constantly increasing. The mounting awareness on that claims for biomarkers of pre-existing disability and frailty [3, 4]. Sclerostin (SOST) is traditionally involved in bone metabolism and associated with frailty and mortality in elderly patients, with recent insights on cardiovascular (CV) risk and immune response [5–7]. Relevance of circulating SOST has been tested in septic patients admitted to intensive care units (ICU) and linked to renal/hepatic organ failure [8]. Besides an increase of 10 pmol/L in serum, SOST has been associated with 30% higher risk of CV mortality in general population [9]. In light of these pleiotropic activities on critical organs, SOST represents a candidate biomarker to encompass the trimodal pattern of sepsis-related mortality. Here, we focused on the role of SOST in a small cohort of elderly septic patients, aiming at preliminarily established its potential role in short- and long-term mortality.

## Materials and methods

### Patient enrollment

The monocentric observational study “Genoa-BASH SEPSIS” enrolled patients between January 2017 and December 2019 at the IRCCS Ospedale Policlinico San Martino in Genoa (Italy). Seventy-three patients were enrolled at the Internal Medicine and Infectious Disease Clinics. The overall follow-up lasted until 31^st^ October 2021. According to the study protocol, all the enrolled patients fulfilled the sepsis-3 defining criteria, as stated by the “Third International Consensus Definition for Sepsis and Septic Shock”: i) suspected infection; ii) acute development of organ dysfunction (within 48 h from enrollment), represented by an increase in the Sequential [Sepsis-related] Organ Failure Assessment (SOFA) score ≥ 2. Quick SOFA (qSOFA) was also calculated as non-diagnostic score with high predictive value for in-hospital mortality outside of the ICU [10].

Sepsis-related exclusion criteria were the start of antibiotic therapy beyond 72 h prior to the enrollment and the inadequate source control within 96 h from the enrollment. Although not standardized, the latter is widely recognized as critical bias influencing treatment of sepsis and restoring of premorbid anatomy and function [11, 12]. Additional exclusion criteria were congestive heart failure with NYHA class III–IV, active cancer (localized, metastatic or under active treatment), hemodialytic treatment, and hepatic cirrhosis. Clinical profiling of enrolled patients included medical history and vital sign assessment. The study was conducted in accordance with the ethical standards of the responsible committee on human experimentation (Regional Ethic Committee approval number 487REG2016 of December 30th, 2016) and with the Helsinki Declaration of 1975. All patients gave informed consent before enrollment.

### Laboratory assay

Blood samples were collected at the day of the enrollment, and then after 7 and 14 days. All samples were then stored at – 80 °C until analysis. Biochemical analyses were performed at central hospital laboratory with routine auto-analyzer and included blood cell count, biochemical assay, and blood gas assay. Circulating levels of SOST were measured at our research laboratory. SOST levels were measured on serum samples obtained from whole blood centrifugation. Colorimetric enzyme-linked immunosorbent assay was performed following manufacturer’s instructions (Quantikine, R&D Systems, Minneapolis, MN). The lower limit of detection for SOST was 31.3 pg/mL with mean intra- and inter-assay coefficients of variation below < 8%, as previously reported [13].

### Study endpoints adjudication and power calculation

Thirty-day mortality has been set as the primary outcome of the present study. Considering the binary outcome of the present study design, our sample size (*n* = 73) does not satisfy the minimum sample size required for developing a clinical prediction model [14]. The results should be then considered as preliminary and deserving validation in further studies. Secondary outcomes of the study were in-hospital and overall mortality rates, also considering SOST levels rise/fall overtime. For these outcomes, patient follow-up has been managed through informatic records. The latter have been defined as for difference (*Δ*) of SOST levels at baseline (T0) with levels at days 7 and 14.

### Statistical analyses

R, version 3.6.3 (R Foundation for Statistical Computing, Vienna, Austria); IBM SPSS Statistics, Version 23.0 (IBM CO., Armonk, NY); and GraphPad Prism 5 (GraphPad Software, Inc, La Jolla, CA) were used for statistical analysis. Categorical data were reported as absolute and relative frequencies and comparisons were drawn by Chi-square or Fisher’s exact test. When the normality assumption—tested by Shapiro–Wilk—was not demonstrated, continuous variables were presented as median and interquartile range (IQR) and intergroup comparisons were drawn by Mann–Whitney *U* test. Likewise, correlations coefficients were calculated by ranked Spearman’s correlation test. Friedman test was instead used for detecting differences in across multiple paired samples. For primary and secondary outcome estimation, we built univariate and adjusted logistic regression models, whose results are reported as odds ratio (OR) and 95% CI. For adjusted models, forward stepwise regression analysis was used, also to avoid overfitting in a quite small cohort. When necessary, variables included in logistic regression model were log-transformed. Model discrimination has been performed by ROC curve analysis, whereas bootstrap resampling performance was used for the internal model validation. Finally, univariate and adjusted Cox proportional hazards model (expressed as hazard ratio [HR] and 95% CI) was built to test overall survival through a forward stepwise regression analysis A two-sided *p* value < 0.05 was considered as statistically significant for all the statistical analyses.

## Results

### Cohort characteristics

Table 1 and Supplementary Tables 1 and 2 summarize clinical and biochemical features of the overall cohort (*n* = 73) at the enrollment and after categorization for the primary outcome.

**Table 1:** Clinical characteristics of the overall cohort at enrollment (n = 73)

	Overall (*n* = 73)	30-day non-survivors (*n* = 17)	30-day survivors (*n* = 56)	*p* value
Age, years [IQR]	82 [76–87]	83 [79–88]	82 [75–87]	0.286
Sex, male (%)	41 (56.2)	13 (73.7)	28 (50.0)	0.098
BMI, Kg/m^2^ [IQR]	25 [21–28]	27 [23–29]	24 [21–27]	0.144
Glasgow coma scale, score [IQR]	15 [13–15]	**13 **[13–15]	**15 **[14–15]	**0.026**
SOFA score [IQR]	3 [2–5]	4 [3–5]	3 [2–4]	0.352
Quick SOFA [IQR]	1 [0–2]	**2 [1–2]**	**1 [0–1]**	**0.001**
Apache II score [IQR]	13 [11–19]	**18 [15–22]**	**13 [10–15]**	**0.005**
Kelly scale [IQR]	1 [1–2]	**2 [2–3]**	**1 [1–2]**	**0.006**
CIRS scale [IQR]	11 [8–15]	**15 [10–20]**	**10 [8–15]**	**0.015**
Charlson comorbidity index [IQR]	3 [1–4]	3 [2–4]	3 [1–4]	0.454
NYHA class of heart failure [IQR]	2 [1–2]	2 [1–2]	2 [1–2]	0.130
Hypertension, *n* (%)	48 (68.6)	10 (62.5)	38 (70.4)	0.532
Diabetes mellitus *n* (%)	19 (27.1)	6 (37.5)	13 (24.1)	0.325
Dyslipidemia, *n* (%)	22 (31.9)	6 (37.5)	16 (30.2)	0.535
COPD, *n* (%)	20 (28.6)	6 (37.5)	14 (25.9)	0.337
IHD, *n* (%)	18 (25.7)	4 (25)	14 (25.9)	1.000
AF, *n* (%)	25 (35.7)	8 (50)	17 (31.5)	0.134
Smoking				
None	24 (42.9)	4 (30.8)	20 (46.5)	0.475
Former, *n* (%)	23 (41.1)	8 (61.5)	15 (34.9)	0.318
Active, *n* (%)	9 (16.1)	1 (7.7)	8 (18.6)	0.659
Packs/year	30 [13–49]	30 [10–30]	30 [20–50]	0.195
Body temperature, °C [IQR]	37.3 [36.5–38.0]	36.8 [36.2–37.6]	37.4 [36.7–38.0]	0.130
HR, bpm [IQR]	84 [73–93]	**94 [80–100]**	**80 [70–90]**	**0.002**
sBP, mmHg [IQR]	115 [100–138]	115 [100–120]	120 [105–140]	0.112
dBP, mmHg [IQR]	70 [60–75]	70 [60 – 79]	70 [60–74]	0.827
RR rate, bpm [IQR]	18 [16–24]	20 [17–24]	18 [16–22]	0.058

Continuous data are presented as median [interquartile range, IQR] whereas categorical ones as absolute (relative) count

The *p* values refer to the comparison between patients deceased and survived within 30 days from the enrollment using the Mann–Whitney test or Chi-square/Fisher’s exact test as appropriate

Significant values are in bold

*BMI* body mass index; *SOFA* sequential organ failure assessment; *CIRS* cumulative illness rating scale; *NYHA* New York Heart Association; *COPD* chronic obstructive pulmonary disease; *IHD* ischemic heart disease; *AF* atrial fibrillation; *HR*: heart rate; *sBP* systolic blood pressure; *dBP* diastolic blood pressure; *RR* respiratory rate

**Fig. 1:**
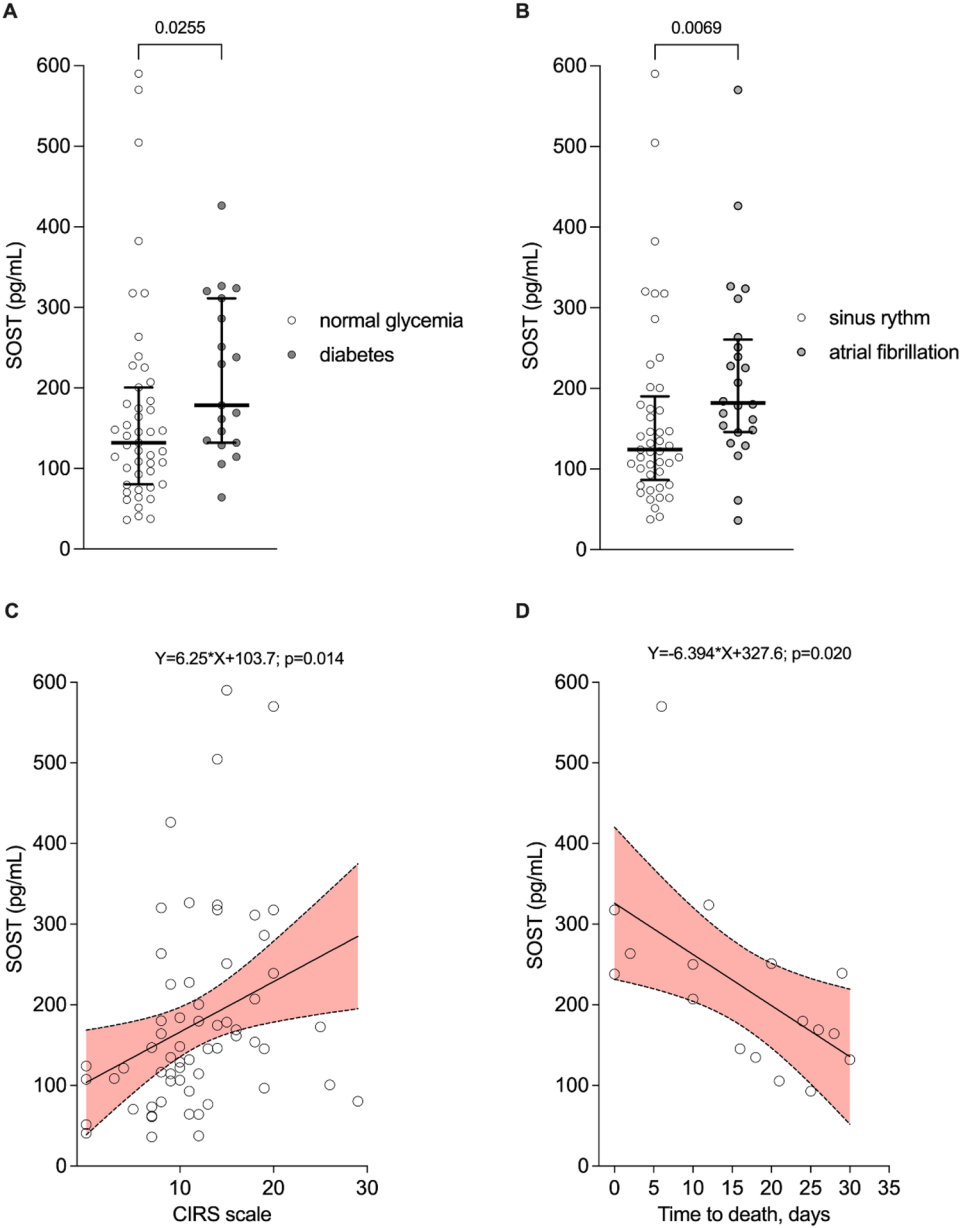
SOST is associated with comorbidity burden and poor survival time. Sclerostin (SOST) distribution across fasting glycemia (**A**) and history of atrial fibrillation (**B**). Regarding continuous variables, SOST shows a positive significant correlation with CIRS scale (**C**) and time to death at 30 days (analysis limited to deceased patients) (**D**)

Patients were very elderly (median age of 82 years), well balanced across sex (56.2% of men), and with relatively low comorbidity burden (median CIRS and Charlson 11 and 3, respectively). As expected, 30-day mortality was associated with a more severe sepsis as expressed by Glasgow coma scale (median 13 *vs.* 15; *p* = 0.013), quick SOFA (median 2 *vs.* 1; *p* = 0.002), APACHE II score (median 18 *vs.* 13; *p* = 0.014), and Kelly scale (median 2 *vs.* 1; *p* = 0.002). Conversely, the comorbidity burden was less relevant, as summarized by the CIRS scale (median 15 *vs.* 10; *p* = 0.042) and the Charlson comorbidity index (*p* = 0.548). Higher heart rate emerged as the most relevant clinical feature associated with 30-day mortality, alongside with anemia (median hemoglobin values 10.1 *vs.* 11.8; *p* = 0.022) and increased circulating levels of SOST (median 193 ng/mL *vs.* 132 ng/mL; *p* = 0.022). Concerning the latter, the most interesting association was with age (*r* = 0.370; *p* = 0.002) and cardiovascular comorbidities: diabetes, ischemic heart disease, and atrial fibrillation (Fig. 1 A and B, Table 2). Accordingly, linear correlations with CIRS scale (*r* = 0.425; *p* < 0.001) and NYHA class (*r* = 0.354; *p* = 0.005) were here reported (Fig. 1 C, Table 3). Further mention deserved the correlation between SOST and body temperature (*r* = – 0.278; *p* = 0.025) and platelet count (*r* = 0.249; *p* = 0.044).

**Table 2:** Baseline sclerostin level variations according to the clinical variables in the overall cohort at enrollment (*n* = 73)

	Sclerostin (ng/mL)	*p* value
Sex		0.053
Men, *n* = 41	129 [93–180]	
Women, *n* = 32	167 [114–260]	
BMI (Kg/m^2^)		0.718
Normal (18.5–24.9)	127 (73–159)	
Overweight (25.0–29.9)	146 (99–239)	
Obese (≥ 30)	145 (106–318)	
Hypertension		0.106
No, *n* = 25	145 [70–180]	
Yes, *n* = 48	146 [114–286]	
Diabetes mellitus		**0.027**
No, *n* = 54	**132 [87–192]**	
Yes, *n* = 19	**178 [133–299]**	
Dyslipidemia		0.855
No, *n* = 51	150 [105–232]	
Yes, *n* = 22	135 [106–228]	
COPD		0.198
No, *n* = 53	138 [96–212]	
Yes, *n* = 20	155 [130–248]	
IHD		0.067
No, *n* = 55	132 [97–201]	
Yes, *n* = 18	178 [141–251]	
AF		**0.007**
No, *n* = 48	**122 [84–174]**	
Yes, *n* = 25	**182 [147–254]**	
Smoker		0.932
No, *n* = 24	145 [107–204]	
Former, *n* = 23	148 [116–228]	
Yes, *n* = 9	138 [96–247]	

Continuous data are presented as median [interquartile range, IQR]

Significant values are in bold

The *p* values were calculated using the Mann–Whitney test or Kruskal–Wallis, as appropriate

*BMI* body mass index; *COPD* chronic obstructive pulmonary disease; *IHD* ischemic heart disease; *AF* atrial fibrillation

**Table 3:** Correlations between baseline SOST and clinical/biochemical variables in the overall cohort

	SOST	
	*r*	*p* value
Age, years	**0.370**	**0.002**
BMI	0.248	0.128
Glasgow coma scale	– 0.057	0.646
Quick SOFA	0.191	0.118
Apache II score	0.231	0.064
Kelly scale	0.043	0.729
CIRS scale	**0.425**	** < 0.001**
Charlson comorbidity index	0.233	0.068
NYHA class of heart failure	**0.354**	**0.005**
Body temperature	**– 0.278**	**0.025**
HR	**– **0.002	0.990
sBP	**– **0.053	0.674
dBP	**– **0.200	0.114
RR rate	0.220	0.085
RBC	**– **0.104	0.399
Hb	**– **0.188	0.124
WBC count	0.163	0.184
PLTs	**0.249**	**0.044**
CRP	**– **0.034	0.783
Procalcitonin	**– **0.069	0.613
Fibrinogen	0.021	0.875
Albumin	**– **0.037	0.803
Creatinine	0.233	0.058
eGFR	**– **0.204	0.128
Glycemia	0.259	0.094
pH	0.061	0.694
pO2	0.098	0.521
pCO2	0.035	0.821
SaO2	0.148	0.337
FiO2	0.049	0.766
HCO3–	**– **0.069	0.657

Significant values are in bold

The *p* values refer to the Spearman's rank correlation coefficients

*BMI* body mass index; *CIRS* cumulative illness rating scale; *CRP* C-reactive protein; *eGFR* estimated glomerular filtration rate; *FiO*_*2*_ fraction of inspired oxygen; *NYHA* New York Heart Association; *Hb* hemoglobin*; HCO*_*3*_ bicarbonate; *HR* heart rate; *sBP* systolic blood pressure; dBP diastolic blood pressure; *pO*_*2*_ partial pressure of oxygen; *pCO*_*2*_ partial pressure of carbon dioxide; *PLT* platelets; *RBC* red blood cells; *RR* respiratory rate; *SaO*_*2*_ arterial oxygen saturation; *SOFA* sequential organ failure assessment; *WBC *white blood cell

### Serum SOST levels predict early death in elderly septic patients

**Fig. 2:**
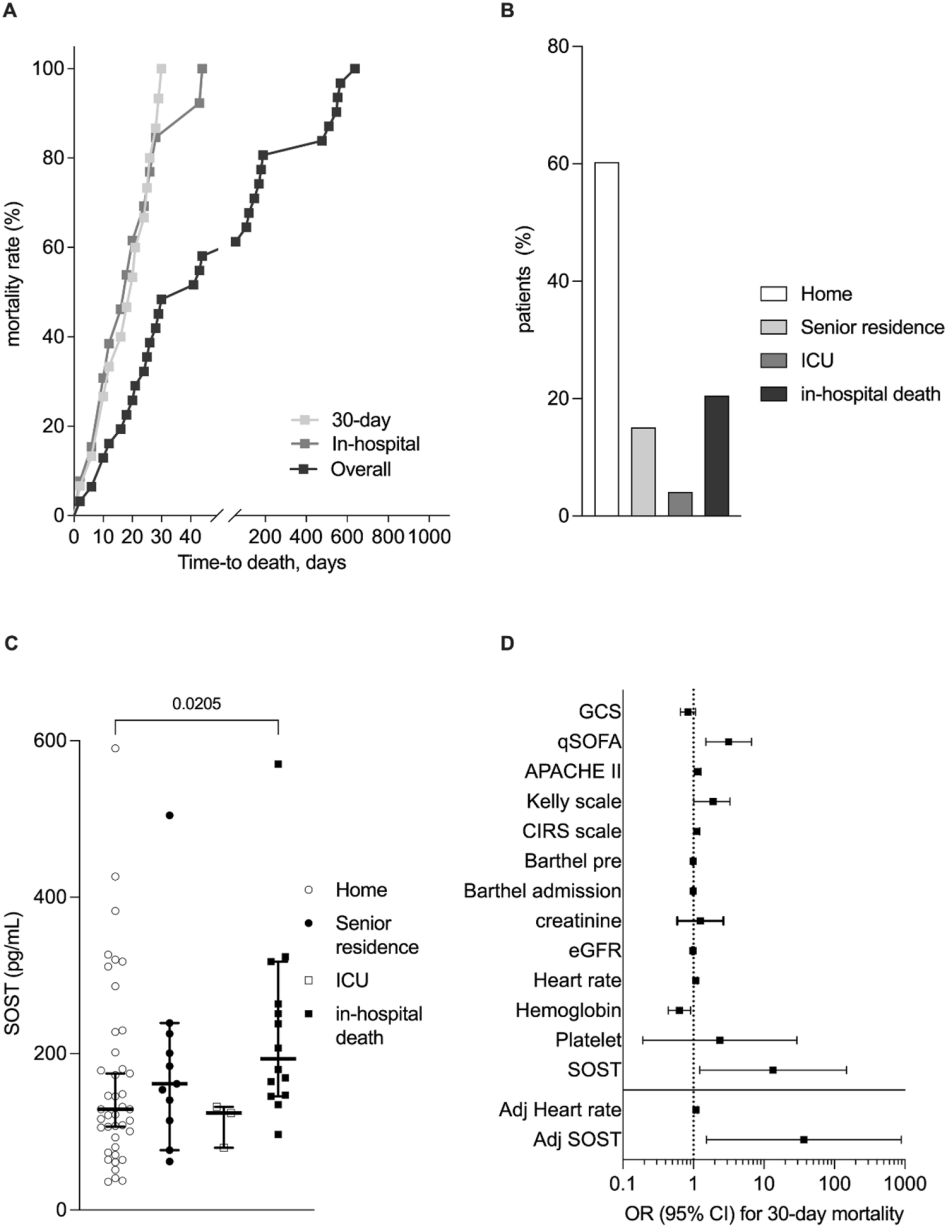
SOST is tightly correlated with early (30 days) death risk. Thirty-four patients (46.6%) deceased during follow-up. Among these, 17 deaths occurred within 30-days from enrollment and 15 during hospitalization (**A**). The latter accounted for 21.9% of patient discharge, whereas others were discharged at home (60.3%), in senior residences (15.1%) or admitted to ICU (4.1%) (**B**). Higher sclerostin (SOST) levels were associated with poor outcome (**C**). At logistic regression, SOST and heart rate were the only significant predictor of 30-day mortality (**D**)

An interesting observation was the significant inverse correlation between SOST and the time to death (*r* = 0.337; *p* = 0.005) (Fig. 1 D). Overall death incidence was 46.6% (*n* = 34) over a median follow-up period of 688 days (range 0 to 1061 days), whereas 30-days mortality occurred in 17 patients (Fig. 2A).

**Fig. 3:**
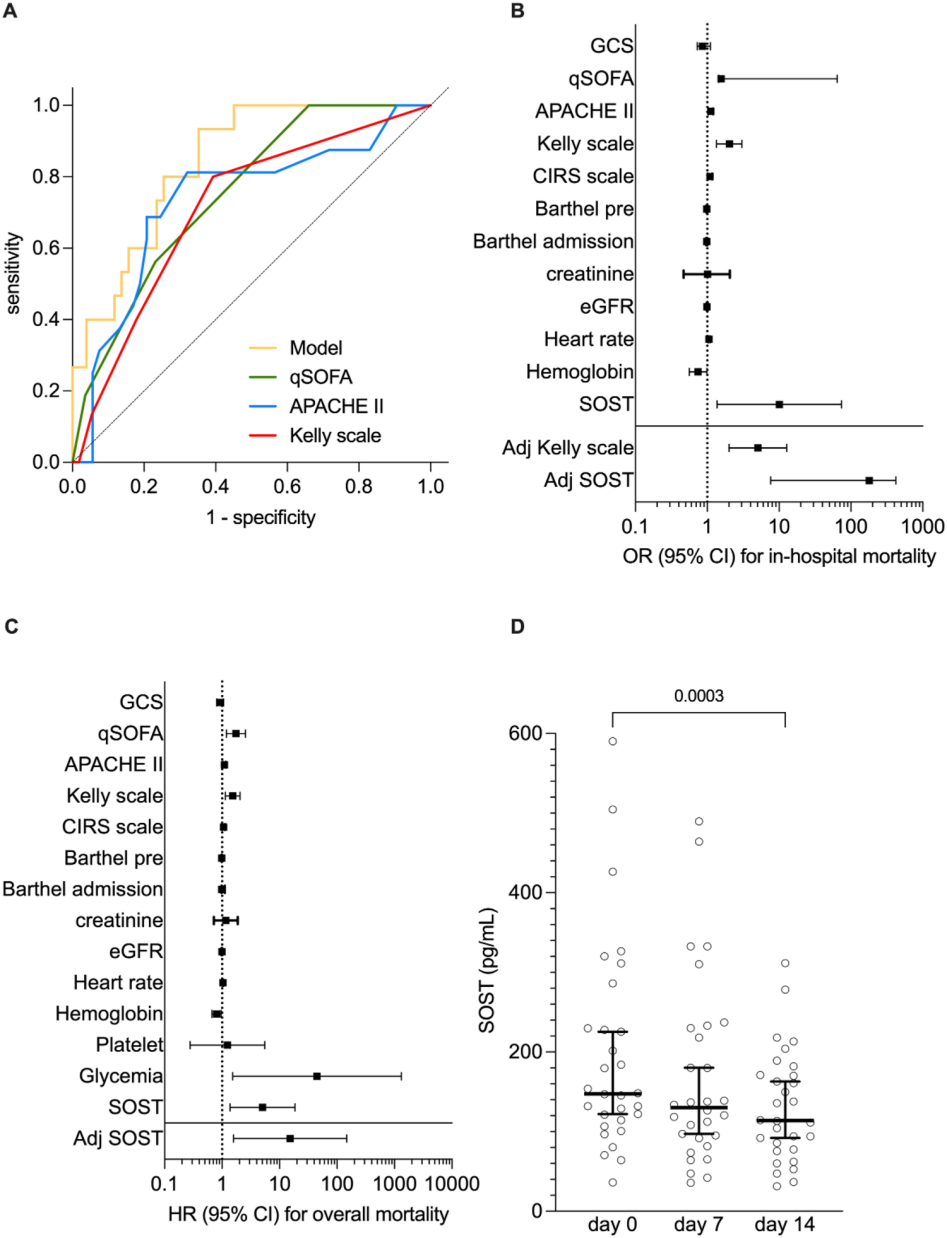
Predictive value of SOST toward mortality. The predictive value of sclerostin (SOST) toward 30-day mortality shows a trend of superiority as compared with toward other validated scores for clinical ill patients (**A**). Such a predictive value of SOST was also confirmed—alongside with Kelly scale—in the Cox regression model for the in-hospital mortality (**B**). SOST shows a predictive value toward long-term all-cause mortality (**C**) and a significant trend to lowering overtime (**D**)

In-hospital death represented the 21.9% of patient discharge and associated with higher baseline serum levels of SOST as compared with patients discharged at home (60.3%), in senior residences (15.1%) or admitted to intensive care unit (ICU) (4.1%) (Fig. 2 B and C). When logistic regression model was built, SOST emerged as predictor of 30-day mortality in septic patients (OR 13.459 with 95% CI 1.226 to 148.017) alongside with heart rate, comorbidity burden assessed by CIRS scale and validated scoring scales for critical ill patients: quick SOFA, APACHE II, Kelly. The forward stepwise regression approach highlighted the independent predictive value of SOST (adjOR 36.887 with 95% CI 1.535 to 886.360) and heart rate (adjOR 1.085 with 95% CI 1.028 to 1.145) (Fig. 2 D; Table S3). With a *p* value of 0.852, the Hosmer–Lemeshow test confirmed the good calibration of this model, whereas the result of ROC curve analysis indicated a good performance with an AUC of 0.825 (Table S5). Although not significant, the model performance was higher than other validated scoring scales for critical ill patients: quick SOFA, APACHE II, Kelly (Fig. 3 A). Internal validation with bootstrap resampling was finally performed. Based on 1000 bootstrap replicates, new estimate of the ORs was obtained (average of the 1000 ORs from the 1000 bootstrap samples). The OR estimated on the original dataset fall within the new bootstrap confidence intervals.

### Serum levels of SOST and secondary outcomes: a wide prediction of mortality in septic patients

The univariate and adjusted Cox proportional hazards regression models for in-hospital mortality confirmed the predictive role of SOST (adjHR 136.191 with 95% CI 6.919 to 2680.871) together with the Kelly scale (adjHR 5.422 with 95% CI 2.305 to 12.754) (Fig. 3 B and Table S4). Serum SOST was also independently associated with long-term mortality with a 15-fold increased risk (adjHR 15.282 with 95% CI 1.5842.106 to 147.463) (Fig. 3 C, Table S5). When overtime modification in circulating SOST levels was considered, a progressive fall at days 7 and 14 after enrollment was observed (Fig. 3 D). Like baseline levels, the extent to which SOST decreased at day 14 was identified as independent predictor of long-term mortality (adjHR for Δ SOST T0 – day 14: 1.009 with 95% CI 1.002 to 1.017) (Table S6).

## Discussion

The major finding of this study is the demonstration of serum SOST as promising mortality predictor in elderly patients with sepsis. Death risk related to SOST levels progressively increases from 5-fold in long-term follow-up up to 10- and 13-fold for in-hospital and 30-day mortality, respectively. Nevertheless, the clinical relevance of SOST in critically ill patients is poorly investigated. A single case–control study has so far reported an association with renal, hepatic, and CV impairment, but not mortality [8]. Here, we confirmed the association with comorbidity burden as expressed by CIRS scale and—partially—Charlson comorbidity index, with special regard to CV risk factors: diabetes, ischemic heart disease, and atrial fibrillation. Predictive value of SOST also prevails on heart rate, widely used alone or within clinical scales [15]. However, we here consider one measure at enrollment, while overtime variability expresses a greater predictive power. Of further interest, the lack of correlation between SOST and qSOFA deserves to be discussed. qSOFA is traditionally described as prognostic scale with greater predictive value than SOFA for in-hospital mortality outside of the ICU [10]. Later clinical studies confirmed the suboptimal diagnostic performance of qSOFA [16–18], even questioning its predictive value [19, 20]. The rate of septic patients with qSOFA values < 2 was here quite higher (46.5%), but in line with recently reported elsewhere [17, 18]. Early sepsis identification in elderly still represents a demanding clinical challenge and accounts for strong efforts in implementing clinical predictive weapons [21–23]. This ultimately represents the rationale of the present study.

As biomarker of bone mineral density, serum levels of SOST may partially reflect the advanced age and frailty of enrolled patients, which are more prone to osteopenia/osteoporosis [24]. Similarly, SOST is implicated in CV health [25] and insulin sensitivity [13] but opposite finding on related adverse outcomes has been reported [9, 26]. Even taking into account the small sample size, there is a tendency to higher SOST levels in female. This seems partially in contrast with previous works.

[27]. We also acknowledge as a study limitation the lack of information about bone mass. Furthermore, the prognostic value of short-term variation in serum SOST may partially overcome this limitation, rather paying attention on the role of SOST as an acute-phase protein [28]. Indirect evidence indeed suggests an active role of SOST in the host immune response to infection. By targeting canonical Wnt signaling, SOST would have a relevant role in self-renewal and differentiation of hematopoietic stem cell within bone marrow niche [29]. Studies on *Sost*^*−/−*^ recipient mice have demonstrated for SOST an inhibitory effect on proliferation and mobilization of myeloid progenitor. These suppression mechanisms would involve lymphoid cell also [30] and potentially platelet count. A positive correlation between SOST levels and platelets count was indeed observed here. Although never reported before, it can be related to the excess of acute-phase response [31]. Furthermore, only one experimental study has so far associated SOST inhibition with increased clearance of platelets and reduction of their count [32, 33].

Metabolic effects on circulating SOST is a topic of further interest. Thirty-day deceased patients showed a tendency toward higher body mass index and prevalence of diabetes. In contrast with previous literature [34, 35], we did not find any significant association between circulating SOST and dysmetabolic status. Furthermore, we have no data on any cardiovascular mortality in the study cohort. They would allow to solve this mismatch and are warranted in further studies.

Over time changes in SOST levels during sepsis and their association with outcomes—mainly time-to-event—further highlight a potential role of SOST as an acute-phase protein. Whereas any association of baseline levels might be related with a pre-existing frailty, such an acute reduction should be viewed as a feedback mechanism preventing a dysregulated host response to infection. In line, high baseline/persistently high SOST levels would sustain mortality risk through a dysregulated host response, in accordance with the third international consensus definitions for sepsis and septic shock (Sepsis-3) [1]. However, this finding needs to be further addressed as short-term changes in SOST levels have not yet been investigated. Rather, long-term changes in SOST levels have been previously reported as marker of aging, but also cardiometabolic health [28, 36, 37].

As additional finding, we set up a long-term follow-up that accomplishes the most recent view on sepsis pathophysiology. There is indeed an increasing awareness about a trimodal pattern of sepsis-related mortality that would last even 3 years after the event. Our findings extend the predictive value of baseline circulating SOST and its overtime variations over a median of 2.5 years. A so long follow-up may raise questions about an actual causal relationship, and this should be acknowledged as additional study limitation. However, the concept of long-term sepsis-related mortality is intrinsically far from any standardization. Advanced age, comorbidity burden, persistent immune dysfunction/inflammation, chronic catabolism, and discharge disposition differently tailor to each patient. Although preliminary and underpowered, SOST may rather reflect a dual role of SOST as a frailty biomarker and an active immune mediator, thus deserving future investigations. Furthermore, our study design gave the opportunity to investigate sepsis and related outcomes in a special setting of patients, poorly considered in previous studies. This field of research is indeed traditionally oriented on critical ill patient admitted to ICU, where patients are somehow selected and largely differ from those admitted in internal medicine wards in terms of median age, comorbidities, standard of care, and outcome. Even the incidence rate of sepsis admitted to internal medicine wards is quite higher (about 367 vs. 44 cases per 100,000 adult/year) [38] and patient heterogeneity is greater as well. The low number of patients enrolled in a long enrollment period may be the expression of this heterogeneity and the clinical challenge to face off. In daily practice, elderly patients admitted to internal medicine wards have a consistent comorbidity burden. When sepsis occurs, their clinical condition rapidly deteriorates and need monitoring at the emergency department. If not started at nursing home, antibiotic treatment is often urgent and empirically based. Far from a standardized approach, it is challenging to recognize an early (within 48 h) increase of SOFA score ≥ 2 in internal medicine wards. Downstream of such a complexity, identifying biomarkers reliable for clinical use is challenged by diversity in assay methods, all-cause mortality as the only outcome, and lack of pathophysiological link among the others [39]. Therefore, studies on biomarkers in sepsis generally do not answer to specific, clinically relevant questions, but rather report just diagnostic or prognostic values. We acknowledge this point as another intrinsic limitation of this unpowered study that should be then considered as pilot. Nevertheless, variations in time course and their prognostic relevance allowed us to hypothesize SOST as not innocent bystander in sepsis.

In conclusion, the present study provides preliminary evidence for a prognostic role of SOST in sepsis. Especially high baseline/persistently higher circulating levels of SOST may predict short- and long-term mortality in elderly patients with sepsis admitted in internal medicine wards. In line with the emerging needs of patients profiling, enhancing recovery from sepsis and get loaded of long-term sequelae, SOST may represent a long-lasting biomarker that is able to guide clinical decisions.

## Supplementary Information

Below is the link to the electronic supplementary material.

Supplementary file1 (DOC 270 KB)
