# Supplementary material for: Early sclerostin assessment in frail elderly patients with sepsis: insights on short- and long-term mortality prediction
Source: Intern Emerg Med. 2023 Mar 21;18(5):1509–19. doi: 10.1007/s11739-023-03223-w (PMC10412666; doi:10.1007/s11739-023-03223-w)
Supplement: Supplementary file 1 — Supplementary file1 (DOC 270 KB) [file 11739_2023_3223_MOESM1_ESM.doc]

**Supplementary Material**

Table S1. Biochemical variables of the overall cohort at enrollment (n=73).

|  | **Overall**  **(n=73)** | **30-day non-survivors**  **(n=17)** | **30-day survivors**  **(n=56)** | **p-value** |
| --- | --- | --- | --- | --- |
| RBC, nx10E12/L [IQR] | 4.1 [3.7 – 4.4] | 3.7 [3.6 – 4.3] | 4.1 [3.7 – 4.5)] | 0.735 |
| Hb, mg/dL [IQR] | 11.6 [10.1 – 12.6] | **10.1 [9.8 – 11.7]** | **11.8 [10.9 – 12.8]** | **0.005** |
| WBC count, nx10E9/L [IQR] | 12.2 [9.2 – 16.5] | 12.5 [10.3 – 19.8] | 12.2 [8.6 – 16.4] | 0.370 |
| PLTs, nx10E9/L [IQR] | 174 [119 – 236] | 237 [143 – 267] | 169 [112 – 208] | 0.243 |
| CRP, mg/L [IQR] | 148 [81 – 199] | 158 [83 – 254] | 145 [81 – 195] | 0.195 |
| Procalcitonin, μg/mL [IQR] | 3.6 [0.5 – 13.2] | 4.2 [0.6 – 12.8] | 3.6 [0.5 – 13.2] | 0.946 |
| Fibrinogen, g/L [IQR] | 5.7 [4.4 – 6.6] | 6.2 [5.2 – 6.8] | 5.4 [4.4 – 6.5] | 0.093 |
| Albumin, g/L [IQR] | 29.8 [27.3 – 33.4] | 26.9 [24.8 – 32.2] | 30.8 [27.9 – 33.4] | 0.037 |
| Creatinine, mg/dL [IQR] | 1.5 [1.0 – 2.0] | 1.9 [1.1 – 2.2] | 1.5 [1.0 – 1.9] | 0.477 |
| eGFR, mL/min [IQR] | 41 [26 – 67] | 25 [23 – 60] | 43 [31 – 72] | 0.079 |
| Glycemia, mg/dL [IQR] | 121 [105 – 148] | 143 [123 – 165] | 120 [102 – 147] | 0.252 |
| SOST ng/mL [IQR] | 146 [107 – 228] | **193 [146 – 254]** | **132 [97 – 201]** | **0.020** |

Continuous data are presented as median [interquartile range, IQR].

The p-values refers to the comparison between patients deceased and survived within 30 days from the enrollment using the Mann-Whitney test.

RBC: red blood cells; Hb: hemoglobin; WBC: white blood cell; PLT: platelets; CRP: C-reactive protein; eGFR, estimated glomerular filtration rate.

Table S2. Arterial blood gas parameters of the overall cohort at enrollment (n=73).

|  | **Overall**  **(n=73)** | **30-day deceased**  **(n=17)** | **30 days survived**  **(n=56)** | **p-value** |
| --- | --- | --- | --- | --- |
| pH | 7.44 [7.40 – 7.48] | 7.45 [7.44 – 7.47] | 7.44 [7.40 – 7.48] | 0.797 |
| pO2, mmHg [IQR] | 61 [51 – 77] | 56 [49 – 91] | 61 [52 – 74] | 0.677 |
| pCO2, mmHg [IQR] | 37 [33 – 43] | 36 [31 – 48] | 37 [33 – 42] | 0.536 |
| SaO2, % [IQR] | 93 [85 – 96] | 92 [87 – 98] | 93 [85 – 96] | 0.241 |
| FiO2, % [IQR] | 21 [21 – 21] | 21 [21 – 21] | 21 [21 – 21] | 0.988 |
| HCO3^–^, mmol/L [IQR] | 25 [22 – 29] | 25 [21 – 32] | 25 [22 – 28] | 0.733 |

Continuous data are presented as median [interquartile range, IQR].

The p-values refers to the comparison between patients deceased and survived within 30 days from the enrollment using the Mann-Whitney test.

pO2: partial pressure of oxygen; pCO2: partial pressure of carbon dioxide; SaO2: arterial oxygen saturation; FiO2: fraction of inspired oxygen; HCO3^–^: bicarbonate.

**Table S3. Independent predictive value of baseline SOST** toward 30-day mortality.

|  | **Univariate** |  | **Adjusted** |  |
| --- | --- | --- | --- | --- |
| **30-day mortality** | **OR (95% CI)** | ***p*-value** | **OR (95% CI)** | ***p*-value** |
| Glasgow Coma Scale | 0.836 (0.654 – 1.069) | 0.153 |  |  |
| **Quick SOFA** | **3.155 (1.500 – 6.640)** | **0.002** |  |  |
| **Apache II Score** | **1.145 (1.029 – 1.274)** | **0.013** |  |  |
| **Kelly scale** | **1.893 (1.088 – 3.294)** | **0.023** |  |  |
| **CIRS scale** | **1.114 (1.010 – 1.230)** | **0.032** |  |  |
| Barthel index pre-admission | 0.989 (0.973 – 1.004) | 0.152 |  |  |
| Barthel index at enrollment | 0.991 (0.973 – 1.009) | 0.316 |  |  |
| Creatinine | 1.254 (0.589 – 2.669) | 0.557 |  |  |
| eGFR | 0.985 (0.959 – 1.011) | 0.249 |  |  |
| **HR** | **1.080 (1.027 – 1.136)** | **0.003** | **1.085 (1.028 – 1.145)** | **0.003** |
| **Hb** | **0.633 (0.440 – 0.910)** | **0.013** |  |  |
| Platelet | 2.367 (0.191 – 29.369) | 0.502 |  |  |
| **SOST** | **13.459 (1.226 – 148.017)** | **0.033** | **36.887 (1.535 – 886.360)** | **0.026** |

Non-normally distributed variables have been Log-transformed. Adjusted model of logistic regression was built by forward stepwise regression approach.

OR: odds ratio; CI: confidence interval; SOFA: sequential organ failure assessment; CIRS, cumulative illness rating scale; HR: heart rate; Hb: hemoglobin.

**Table S4. Independent predictive value of baseline SOST** toward in-hospital mortality.

|  | **Univariate** |  | **Adjusted** |  |
| --- | --- | --- | --- | --- |
| **In-hospital mortality** | **OR (95% CI)** | ***p*-value** | **OR (95% CI)** | ***p*-value** |
| Glasgow Coma Scale | 0.859 (0.724 – 1.018) | 0.080 |  |  |
| **Quick Sofa** | **1.564 (1.446 – 64.548)** | **0.001** |  |  |
| **Apache II Score** | **1.123 (1.045 – 1.207)** | **0.002** |  |  |
| **Kelly scale** | **2.032 (1.346 – 3.029)** | **<0.001** | **4.984 (2.174 – 11.427)** | **<0.001** |
| **CIRS scale** | **1.091 (1.013 – 1.174)** | **0.021** |  |  |
| **Barthel index pre-admission** | **0.985 (0.971 – 1.000)** | **0.048** |  |  |
| Barthel index at enrollment | 0.982 (0.963 – 1.002) | 0.081 |  |  |
| Creatinine | 1.012 (0.496 – 2.066) | 0.973 |  |  |
| eGFR | 0.993 (0.970 – 1.016) | 0.550 |  |  |
| Body temperature | 0.047 (0.001 – 3.823) | 0.173 |  |  |
| **Heart rate** | **1.059 (1.028 – 1.092)** | **0.014** |  |  |
| **Hemoglobin** | **0.746 (0.563 – 0.989)** | **0.042** |  |  |
| **SOST** | **10.089 (1.375 – 74.013)** | **0.023** | **172.023 (9.723 – 3043.539)** | **<0.001** |

Non-normally distributed variables have been Log-transformed. Adjusted model of Cox regression was built by forward stepwise regression analysis.

OR: odds ratio; CI: confidence interval; SOFA: sequential organ failure assessment; CIRS: cumulative illness rating scale.

**Table S5. Independent predictive value of baseline SOST toward overall mortality at follow-up.**

|  | **Univariate** |  | **Adjusted** |  |
| --- | --- | --- | --- | --- |
| **Overall mortality** | **HR (95% CI)** | ***p*-value** | **HR (95% CI)** | ***p*-value** |
| Glasgow Coma Scale | 0.927 (0.808 – 1.064) | 0.283 |  |  |
| Quick Sofa | **1.742 (1.189 – 2.553)** | **0.004** |  |  |
| Apache II Score | **1.097 (1.038 – 1.160)** | **0.001** |  |  |
| Kelly scale | **1.529 (1.144 – 2.045)** | **0.004** |  |  |
| CIRS scale | **1.065 (1.012 – 1.120)** | **0.016** |  |  |
| Barthel index pre-admission | **0.989 (0.979 – 0.998)** | **0.017** |  |  |
| Barthel index at enrollment | 0.989 (0.978 – 1.110) | 0.070 |  |  |
| Creatinine | 1.163 (0.719 – 1.882) | 0.539 |  |  |
| eGFR | 0.991 (0.976 – 1.005) | 0.211 |  |  |
| Heart rate | **1.033 (1.007 – 1.059)** | **0.014** |  |  |
| Hemoglobin | **0.802 (0.669** – **0.962)** | **0.018** |  |  |
| Platelet | 1.238 (0.277 – 5.521) | 0.780 |  |  |
| Glycemia | **44.845 (1.532 – 1313.010)** | **0.027** |  |  |
| SOST | **5.061 (1.379 – 18.570)** | **0.014** | **15.282 (1.584 – 147.463)** | **0.018** |

Non-normally distributed variables have been Log-transformed. Adjusted model of Cox regression was built by forward stepwise regression analysis. OR: odds ratio; CI: confidence interval; HR: hazard ratio; CI: confidence interval; CIRS: cumulative illness rating scale.

**Table S6. Cox regression for overall mortality at follow-up.**

|  | **Univariate** |  | **Adjusted** |  |
| --- | --- | --- | --- | --- |
| **Overall mortality** | **HR (95% CI)** | ***p*-value** | **HR (95% CI)** | ***p*-value** |
| Glasgow Coma Scale | 0.927 (0.808 – 1.064) | 0.283 |  |  |
| Quick Sofa | **1.742 (1.189 – 2.553)** | **0.004** |  |  |
| Apache II Score | **1.097 (1.038 – 1.160)** | **0.001** |  |  |
| Kelly scale | **1.529 (1.144 – 2.045)** | **0.004** |  |  |
| CIRS scale | **1.065 (1.012 – 1.120)** | **0.016** |  |  |
| Barthel index pre-admission | **0.989 (0.979 – 0.998)** | **0.017** |  |  |
| Barthel index at enrollment | 0.989 (0.978 – 1.110) | 0.070 |  |  |
| Creatinine | 1.163 (0.719 – 1.882) | 0.539 |  |  |
| eGFR | 0.991 (0.976 – 1.005) | 0.211 |  |  |
| Heart rate | **1.033 (1.007 – 1.059)** | **0.014** |  |  |
| Hemoglobin | **0.802 (0.669** – **0.962)** | **0.018** |  |  |
| Platelet | 1.238 (0.277 – 5.521) | 0.780 |  |  |
| Glycemia | **44.845 (1.532 – 1313.010)** | **0.027** |  |  |
| Δ SOST T0 – day 14 | **1.006 (1.001 – 1.011)** | **0.024** | **1.009 (1.002 – 1.017)** | **0.012** |

| **Section/Topic** | **Item** | **Checklist Item** | **Page** |
| --- | --- | --- | --- |
| **Title and abstract** | | | |
| Title | 1 | Identify the study as developing and/or validating a multivariable prediction model, the target population, and the outcome to be predicted. | 1 |
| Abstract | 2 | Provide a summary of objectives, study design, setting, participants, sample size, predictors, outcome, statistical analysis, results, and conclusions. | 2 |
| **Introduction** | | | |
| Background and objectives | 3a | Explain the medical context (including whether diagnostic or prognostic) and rationale for developing or validating the multivariable prediction model, including references to existing models. | 3-4 |
|  | 3b | Specify the objectives, including whether the study describes the development or validation of the model or both. | 3-4 |
| **Methods** | | | |
| Source of data | 4a | Describe the study design or source of data (e.g., randomized trial, cohort, or registry data), separately for the development and validation data sets, if applicable. | 4-5 |
|  | 4b | Specify the key study dates, including start of accrual; end of accrual; and, if applicable, end of follow-up. | 4 |
| Participants | 5a | Specify key elements of the study setting (e.g., primary care, secondary care, general population) including number and location of centres. | 4-5 |
|  | 5b | Describe eligibility criteria for participants. | 4 |
|  | 5c | Give details of treatments received, if relevant. | 4 |
| Outcome | 6a | Clearly define the outcome that is predicted by the prediction model, including how and when assessed. | 4 |
|  | 6b | Report any actions to blind assessment of the outcome to be predicted. |  |
| Predictors | 7a | Clearly define all predictors used in developing or validating the multivariable prediction model, including how and when they were measured. | 4 |
|  | 7b | Report any actions to blind assessment of predictors for the outcome and other predictors. | - |
| Sample size | 8 | Explain how the study size was arrived at. | 5 |
| Missing data | 9 | Describe how missing data were handled (e.g., complete-case analysis, single imputation, multiple imputation) with details of any imputation method. | - |
| Statistical analysis methods | 10a | Describe how predictors were handled in the analyses. | 5-6 |
|  | 10b | Specify type of model, all model-building procedures (including any predictor selection), and method for internal validation. | 5 |
|  | 10d | Specify all measures used to assess model performance and, if relevant, to compare multiple models. | 5-6 |
| Risk groups | 11 | Provide details on how risk groups were created, if done. |  |
| **Results** | | | |
| Participants | 13a | Describe the flow of participants through the study, including the number of participants with and without the outcome and, if applicable, a summary of the follow-up time. A diagram may be helpful. | 6-7 |
|  | 13b | Describe the characteristics of the participants (basic demographics, clinical features, available predictors), including the number of participants with missing data for predictors and outcome. | 4,6; T1-T3 |
| Model development | 14a | Specify the number of participants and outcome events in each analysis. | 7; T1 |
|  | 14b | If done, report the unadjusted association between each candidate predictor and outcome. | 7; T8 |
| Model specification | 15a | Present the full prediction model to allow predictions for individuals (i.e., all regression coefficients, and model intercept or baseline survival at a given time point). | 7; T8 |
|  | 15b | Explain how to the use the prediction model. | 9-10 |
| Model performance | 16 | Report performance measures (with CIs) for the prediction model. | 7; T8 |
| **Discussion** | | | |
| Limitations | 18 | Discuss any limitations of the study (such as nonrepresentative sample, few events per predictor, missing data). | 10 |
| Interpretation | 19b | Give an overall interpretation of the results, considering objectives, limitations, and results from similar studies, and other relevant evidence. | 8-10 |
| Implications | 20 | Discuss the potential clinical use of the model and implications for future research. | 9-10 |
| **Other information** | | | |
| Supplementary information | 21 | Provide information about the availability of supplementary resources, such as study protocol, Web calculator, and data sets. | - |
| Funding | 22 | Give the source of funding and the role of the funders for the present study. | - |

We recommend using the TRIPOD Checklist in conjunction with the TRIPOD Explanation and Elaboration document.

# Table S5 Model discrimination

| **Variable** | **AUC** | **p-value** |
| --- | --- | --- |
| Model | 0.825 | Ref |
| qSOFA | 0.758 | 0.259 |
| APACHE II | 0.702 | 0.151 |
| Kelly scale | 0.737 | 0.259 |

p-values refer to comparisons with the model identified by the stepwise regression approach on logistic regression

**Bootstrap resampling performance.**

Based on 1000 bootstrap replicates, we obtained the following estimate of the OR (average of the 1000 ORs from the 1000 bootstrap samples) and of their 95% confidence interval. When the OR estimated from the original dataset are considered, we note that they are similar to the new bootstrap confidence intervals.

SOST OR 30.223 (1.329 – 687.293); p=0.010

Heart rate OR 1.086 (1.031 – 1.144); p=0.001

**Sample size considerations**

Considering this study as a model with binary outcome, and relying on <https://www.bmj.com/content/368/bmj.m441>, our sample size (**n=73**) does not satisfied the minimum sample size required for: i) a 95% confidence interval for the overall outcome proportion of 0.5 (**n≥383**); ii) a Mean Absolute Precision Error (MAPE) < 0.05 (**n≥129**); iii) an expected uniform shrinkage factor <10% (**n≥169**).
